# Efficacy and Safety of Chinese Herbal Formula Granules in Treating Chronic Kidney Disease Stage 3: A Multicenter, Randomized, Placebo-Controlled, Double-Blind Clinical Trial

**DOI:** 10.1155/2020/4073901

**Published:** 2020-10-21

**Authors:** Jing Zhao, Wei Sun, Jihong Chen, Zhuxing Sun, Dai Chen, Chunhua Cao, Min Yang, Jipei Ma, Ling Wang, Changying Xing, Yan Chen, Meixiao Sheng, Enchao Zhou, Lingdong Xu, Kun Gao, Lihua Liu, Qiong Liu, Lan Yi, Weiming He, Yuanyuan Zhu

**Affiliations:** ^1^Department of Nephrology, Affiliated Hospital of Nanjing University of Chinese Medicine, Nanjing, Jiangsu, China; ^2^Department of Nephrology, Wuxi People's Hospital, Wuxi, Jiangsu, China; ^3^Department of Nephrology, Changzhou TCM Hospital, Changzhou, Jiangsu, China; ^4^Department of Nephrology, Taizhou Hospital of TCM, Taizhou, Zhejiang, China; ^5^Department of Nephrology, The First People's Hospital of Changzhou, Changzhou, Jiangsu, China; ^6^Department of Nephrology, Wuxi Hospital of TCM, Wuxi, Jiangsu, China; ^7^Department of Nephrology, Xuzhou No.1 People's Hospital, Xuzhou, Jiangsu, China; ^8^Department of Nephrology, Jiangsu Province Hospital, Nanjing, Jiangsu, China; ^9^Department of Nephrology, Jiangsu Province Official Hospital, Nanjing, Jiangsu, China

## Abstract

**Background:**

It is generally considered that traditional Chinese medicine (TCM) therapy postpones the progression of some chronic kidney diseases (CKDs). Chinese medicine herbs are widely applied in TCM therapy. We aimed to evaluate clinical efficacy and safety of Chinese herbal formula granules in patients with CKD stage 3 through a prospective randomized controlled study.

**Methods:**

A total of 343 participants with CKD stage 3 were recruited from 9 hospitals in Jiangsu Province between April 2014 and October 2016. Participants were randomly assigned to a treatment or control group. Patients in the treatment group orally took Chinese herbal formula granules twice a day, while controls received placebo granules. The duration of intervention was 24 weeks. Primary outcomes were 24-hour proteinuria, serum creatinine, and eGFR, which were measured every 4 weeks.

**Results:**

There was no statistical difference in 24-hour proteinuria between the two groups (0.97 ± 1.14 g/d vs. 0.97 ± 1.25 g/d). Patients in the treatment group had significantly lower serum creatinine level (130.78 ± 32.55 *μ*mol/L versus 149.12 ± 41.27 *μ*mol/L) and significantly higher eGFR level (55.74 ± 50.82 ml/min/1.73·m^2^ versus 44.46 ± 12.60 ml/min/1.73·m^2^) than those in the control group (*P* < 0.05). There was no significant difference between two groups in the incidence of adverse events.

**Conclusion:**

The treatment adopting Chinese herbal formula granules for 24 weeks improved kidney function of patients with CKD stage 3.

## 1. Introduction

Chronic kidney disease (CKD) stands for a health threat around the world. Its prevalence is between 8% and 16% across regions and locates at around 11% among developed nations such as America and Australia [1]. With the exacerbation in its prevalence and relevant economic burden, this disease attracts accumulating attentions [2]. In our country, CKD prevalence among middle-age and elderly people achieves 11.5%, and merely, 8.7% of these cases are diagnosed while 4.7% experience proper treatments [3]. Some subtypes of this disease would evolve into end-stage renal disease (ESRD), ultimately leading to the implementation of kidney replacement. Until now, over 2.60 million cases have received renal replacement, when more than 2.28 million cases would have been dead if without such therapy [4]. Statistics in the Chinese Renal Data System, a national registry system recording information on patients undergoing dialysis, show that glomerular disease still represents a predominant reason for ESRD (57.4%), followed by diabetic nephropathy (16.4%), hypertension (10.5%), and cystic kidney disease (3.5%) [5]. Fundamental principle for treating CKD lies in postponing ESRD onset. Unfortunately, available therapeutic options are limited, and no measures could definitely protract the progression [6]. How to effectively achieve this goal becomes a grave challenge for global nephrologists in health care systems.

CKD stage 3 is essential in the disease course, showing high morbidity rate. Nearly 11.1% of CKD cases in America are categorized into stages 1–3, with half of them in stage 3, and the number of sufferers in this group expands every year [7]. CKD stage 3 refers to a status between early and middle phases during the whole course of the disease and could be further classified into stages 3a and 3b [8]. Diagnosis and therapy for this stage would be vital for subsequent advancement and outcome of the disease.

Traditional Chinese medicine (TCM) has a history of more than 2500 years in China. TCM therapy includes herbal medicine, acupuncture, tui na (massage), qigong (exercise), and dietary therapy. Among them, herbal medicine has been studied intensively. Ancient people developed many treatments for edema and hematuria, common symptoms of kidney disease. Chinese medicine herbs have been proven to be effective in treating kidney diseases in both ancient and modern times. With medical progressions, more and more studies have checked therapeutic effects of TCM, especially herbs and herbal compounds, in treating CKD [9–11]. It has been reported that some traditional Chinese herbs could reduce proteinuria, protect podocyte, and alleviate glomerulosclerosis [12, 13]. However, relevant clinical studies are rare, with poor quality. Besides, it is difficult to ascertain the efficacy and mechanism of TCM herb compound formula because of diverse active constituents and formula uniformity. TCM focuses not only on kidney injury, but also on internal balance of the whole body. A personalized herbal decoction is based on accurate identification of syndromes, the establishment of therapeutic method, and appropriate choice of herbs and doses. TCM physician will prescribe a personalized herbal formula based on a patient's own conditions, which may repair damaged kidney tissues, improve kidney functions, and alleviate symptoms naturally. Different herbs show varied natural abilities in kidney management. For clinical practice, high quality and well-designed clinical studies would be urgently needed to ascertain the efficacy and safety of Chinese herbs.

Our previous study suggested that Chinese herbal formula granules could retard the increase of serum creatinine in early- and middle-stage CKD [14–16]. However, detailed efficacy and safety of this herbal formula need to be further studied. Accordingly, a prospective, multicenter, randomized, placebo-controlled, double-blind clinical trial was conducted to further verify the efficacy and safety of the Chinese herbal formula in treating CKD stage 3.

## 2. Methods

### 2.1. Study Design

This study was an investigator-initiated, multicenter, double-blind, randomized clinical trial to analyze the efficacy and safety of herbs in treating stage 3 CKD. Patients in the treatment group received Chinese medicine herbs plus basic care, while those in the control group were treated with placebo plus basic care. The study was conducted in 9 centers across Jiangsu Province in China, including Affiliated Hospital of Nanjing University of Chinese Medicine, Wuxi People's Hospital, Changzhou TCM Hospital, Taizhou Hospital of TCM, the First People's Hospital of Changzhou, Wuxi Hospital of TCM, Xuzhou No.1 People's Hospital, Affiliated Hospital of Nanjing Medical University, and Jiangsu Province Official Hospital. This study was approved by the ethics committee of Affiliated Hospital of Nanjing University of Chinese Medicine (approval number: AF/SQ140303). Full trial protocol can be accessed from this hospital.

### 2.2. Study Population

Recruited male and female patients were diagnosed with primary CKD and aged 18–75 years, with estimated glomerular filtration rate (eGFR) ≥ 30 ml/min/1.73 m^2^ and <60 ml/min/1.73 m^2^ which was calculated with chronic kidney disease epidemiology collaboration (CKD-EPI) equation and 24-hour proteinuria ≤2.0 g/d. Blood pressure was managed ≤130/80 mmHg. Patients with acute kidney injury, polycystic kidney disease, or secondary kidney diseases such as diabetic nephropathy were not recruited. Patients receiving glucocorticoids, immunosuppressants, and/or *Tripterygium* drug were excluded. Patients with concurrent serious diseases, including primary cardiac, cerebral, pulmonary, hepatic, hematological, and psychiatric disease, in pregnancy or in lactation, were not included either. All participants signed written informed consent before enrollment.

### 2.3. Data Collection and Monitoring

Case report forms were filled, maintained by an appropriately qualified physician, and checked by data monitor for integrity and accuracy. The monitor reviewed all study records on a case-by-case basis and completed Monitor Review Form. All data were documented into Medroad data acquisition system using double-data entry. The data acquisition system was developed by the Jiangsu Famous Medical Technology Co., Ltd.

### 2.4. Interventions

#### 2.4.1. Drugs, Doses, and Placebo

Chinese herbal formula granules were manufactured by Jiangyin Tianjiang Pharma (Jiangsu, China) and consisted of 10 Chinese herbs as shown in Table 1. Each of the adopted Chinese herbs was supplied in granulate form and packaged in individual bags. Simulated granules containing 10 Chinese herb matching placebo granules were also manufactured by Jiangyin Tianjiang Pharma. Each of those matching placebo granules contained 2.5% of corresponding Chinese herb, was granulated with dextrin to achieve the same total quantity, and then packaged after color and taste adjustments.

The participants in the treatment group were instructed to mix 10 Chinese herbal granules, 20 g in total, dissolve them in 150 ml warm water every time, and drink twice a day. For the control group, 10 matching placebo granules were mixed (20 g), dissolved in 150 ml warm water, and drunk twice a day.

### 2.5. Treatment

Patients received herbal formula granules and standard medical care in the treatment group, and those in the control group took placebo plus standard medical care. Treatment was continued for 24 weeks. All participants received the following standard medical cares, namely, nutritional support and appropriate medications to control blood pressure and lipids and to correct acid-base balance. Participants adopted low-protein diet to maintain a protein intake of 0.6–0.8 g/kg·d with a high biological value >50% and a caloric intake of 30–35 kcal/kg·d. According to the recommended standards outlined in the Seventh Report of the Joint National Committee on the Prevention, Detection, Evaluation, and Treatment of High Blood Pressure (JNC VII) and Kidney Disease Outcome Quality Initiative (K/DOQI) guidelines, angiotensin-converting enzyme inhibitors (ACEIs) or angiotensin II receptor blockers (ARBs) were preferred in controlling blood pressure, and other antihypertensive drugs such as calcium channel blockers would be added for patients whose blood pressure was more than 130/80 mmHg. As for agents regulating blood lipid, statins were preferred with a total cholesterol target <5.72 mmol/L (<220 mg/dl), LDL cholesterol < 0.364 mmol/L (<140 mg/dl), and triglyceride < 2.26 mmol/L (<200 mg/dl).

### 2.6. Outcome Assessment

Primary efficacy outcomes were estimated via changes in 24-hour proteinuria, serum creatinine, and eGFR, and the indicators were measured every 4 weeks for 24 weeks. The participants were instructed to collect urine with an interval over 24 hours (from 7:00 AM of the first day to 7:00 AM of the next day) using a study-specific container, record collection time, and total volume of urine, and then bring urine samples to the corresponding study hospital for the measurement of 24-hour proteinuria determined through the methods of dye-binding (Coomassie Brilliant Blue G-250) using cerebrospinal fluid protein test kit. Blood samples were collected to examine serum creatine (Determiner L CRE kit) and to calculate eGFR.

### 2.7. Safety

Safety evaluation involved participants' general conditions, incidence of adverse events, and laboratory indexes (from hematology and liver function tests and ECGs). Potential adverse events included gastrointestinal reactions, electrolyte disturbances, and abnormal liver function. Events were recorded and assessed every 4 weeks from the start to the end of the study. Once an adverse event occurred during the study, appropriate evaluation and medications were taken according to its severity. Serious adverse events were monitored up to 30 days after the final visit. Participants withdrawing from the trial due to adverse events were followed until the events were settled, and their statuses were documented in detail.

### 2.8. Follow-up Measurements

Participants were followed up every 4 weeks to assess their clinical condition, blood pressure, adverse events, and therapy adherence. 24-hour proteinuria was measured every 4 weeks during the 24-week trial, and blood samples were obtained at 0, 4, 12, and 24 weeks for measuring hemoglobin, red blood cell, white blood cell, platelet, alanine aminotransferase, aspartate aminotransferase, serum urea nitrogen, serum creatinine, serum albumin, and blood lipids. For measuring blood pressure, patients seated quietly for at least 5 minutes, relaxed, and did not move or speak before measurements. Their arms were placed at a level equal to that of their hearts, without constrictions by tight clothing. Measurements were implemented twice for each patient with a mercury sphygmomanometer, and average values were recorded for analysis.

### 2.9. Sample Size Determination

According to the published literature and previous clinical data, sample size was estimated at 300 subjects on the basis of a type I error rate of 0.049 and type II error (*β*) of 0.1, which contributed to the *F* (*α*, *β*) of 10.5 according to the F-distribution table. Assuming a dropout rate no more than 20% for each site, an additional 20% samples should be added, and sample size would be expected to be 360 in total. The formula for the number of subjects was as follows:

(1) $$\begin{array}{c} \text{Number of subjects}=\left\{\left[P1\times \left(100-P1\right)+P2\times \left(100-P2\right)\right]/\left(P2-P1\right)\right\}\times f\left(\alpha ,\beta \right). \end{array}$$

### 2.10. Randomization and Masking

Block randomization scheme was used and stratified by site. Random sequence stratified by site was randomly generated adopting the SAS system. Consecutive numbers were assigned to each site, and the subjects were randomly assigned in the order of their enrollment into the study, with a 1 : 1 ratio to receive Chinese herbal formula granules (the treatment group) or placebo (the control group). Patients, investigators, and site staff were blind to treatment assignment throughout the study.

### 2.11. Statistical Analysis

SAS software v9.2 (SAS Institute Inc., NC, USA) was used for all statistical analyses. All statistical tests were two-sided, and *P* < 0.05 was considered to represent the presence of statistical significance. Descriptive statistics on quantitative variables were described as the number of observations, mean, standard deviation, median, minimum, and maximum. The number of observations and percentages were recorded for categorical variables. Comparisons on quantitative variables between treatment and control groups were performed through grouped *T*-test, while the chi-square test was adopted to compare categorical variables. Meanwhile, repeatedly measured data on 24-hour proteinuria, creatinine, nitrogen, etc. were compared, and their differences were analyzed by variance analyses of repeated measurement. Baseline was defined as the screening period. For parameters not measured at baseline, screening values were used.

## 3. Results

### 3.1. Follow-up

**Figure 1:**
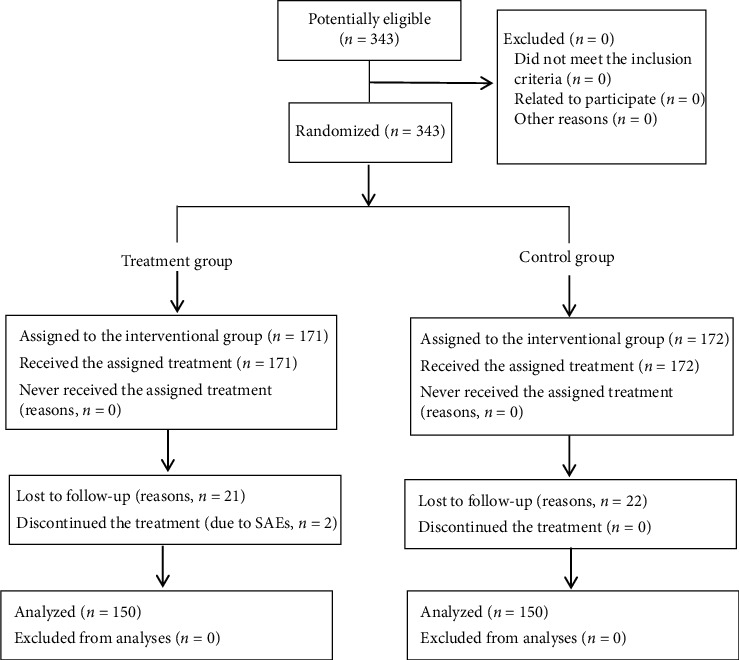
Trial flowchart.

From April 2014 to October 2016, a total of 343 patients with CKD stage 3 were enrolled from 9 centers and randomly assigned into the treatment group (*n* = 171) and control group (*n* = 172). 300 participants completed the 24-week follow-up. 21 subjects were deleted from the treatment group, including 2 subjects with serious adverse events, while 22 from the control group (Figure 1). Full analysis set (FAS) was performed in all patients. Per-protocol set (PPS) was 87.72% in the treatment group and 87.21% in the control group and was 87.46% for the total enrolled participants (Table 2). There were no statistically significant differences in safety profile, dropout rate, or patient compliance between the two groups (Table 3).

### 3.2. Baseline Characteristics

Gender, age, disease course, blood pressure, and the levels of 24-hour proteinuria, eGFR, urea nitrogen, and serum creatine were compared between treatment and control groups before treatment. The results showed that there was no significant difference between two groups in any characteristics at baseline (*P* > 0.05, Table 4).

### 3.3. Outcome Evaluation

#### 3.3.1. Evaluation of 24-Hour Proteinuria

There was no statistical difference in 24-hour proteinuria between the treatment and control groups at the end of treatment (24 weeks later). However, percentage changes in proteinuria were slight at week 8, 12, 16, and 20 in the treatment group, and differences in 24-hour proteinuria before and after treatment were not statistically significant, according to pair-wise comparisons. In contrast, differences in the proteinuria level before and after drug administration were statistically significant in the control group at week 12, 16, 20, and 24 (*P* < 0.05, Table 5).

#### 3.3.2. Serum Creatinine

**Figure 2:**
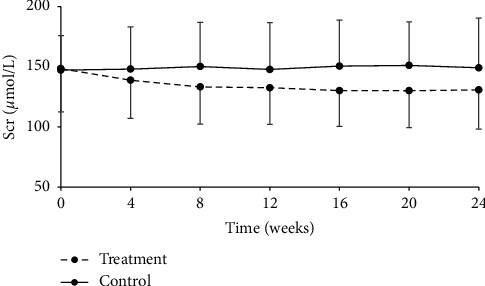
Change from baseline in serum creatinine over the 24-week follow-up period.

Serum creatinine was gradually decreased from the start of treatment and such trend continued throughout the treatment period in the treatment group, showing significant difference at week 16, 20, and 24 (*P* < 0.05); while this index remained stable in the control group (*P* > 0.05). Moreover, statistically significant difference was found between the two groups after drug administration (*P* < 0.001, Table 6, Figure 2). At the final visit, the median change in serum creatinine level before and after treatment was −13.15 *μ*mol/L (range: −42.05–40.77 *μ*mol/L) in the treatment group and 0.48 *μ*mol/L (range: −96.73–320.82 *μ*mol/L) in the control group. *P* value for grouped *T*-test was less than 0.0001, indicating that renal function was improved and therapeutic efficacy in the treatment group was obviously better than that in the control group (Table 7).

#### 3.3.3. Subgroup Analysis

**Figure 3:**
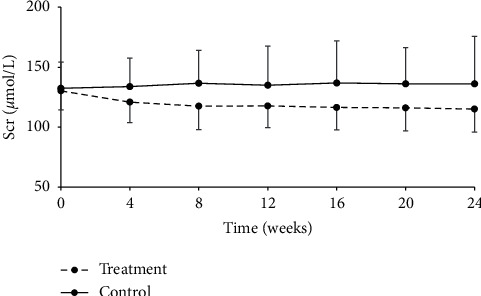
Change from baseline in serum creatinine over the 24-week follow-up period in the CKD stage 3a subgroup, *P* < 0.001.

**Figure 4:**
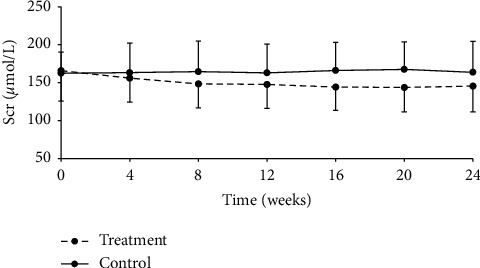
Change from baseline in serum creatinine over the 24-week follow-up period in the CKD stage 3b subgroup, *P* < 0.001.

The participants were further divided into CKD stage 3a (eGFR 45–59 ml/min/1.73 m^2^) and stage 3b (eGFR 30–44 ml/min/1.73 m^2^) groups. As a result, 153 patients with CKD stage 3a (75 in the treatment group and 78 in the control group) were analyzed. Serum creatinine level in the treatment group started to decline from week 4 after treatment, and such tendency maintained throughout the rest of the treatment period. Differences between two groups were statistically significant at all timepoints after treatment (*P* < 0.001, Table 8, Figure 3). 180 subjects were in stage 3b (92 in the treatment group and 88 in the control group). Significant differences were also found in serum creatinine level between the treatment and control groups at any detection time points after drug administration (*P* < 0.001, Table 9, Figure 4).

#### 3.3.4. eGFR

**Figure 5:**
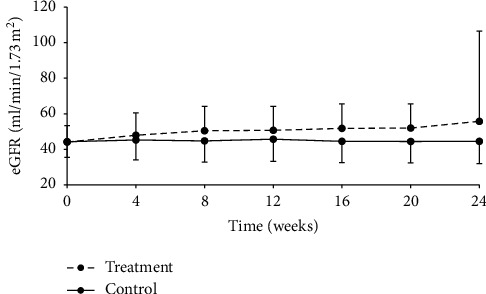
Change from baseline in eGFR over the 24-week follow-up period.

At week 24, eGFR was 55.74 ± 50.82 ml/min/1.73 m^2^ and 44.46 ± 12.60 ml/min/1.73·m^2^ in treatment and control groups, respectively. The difference between groups was statistically significant, with *P* < 0.0001 (Table 10, Figure 5). Statistical analysis indicated that eGFR changes were 17.95 ± 21.86 ml/min/1.73·m^2^ and 0.04 ± 19.83 ml/min/1.73 m^2^ in the treatment and control groups, respectively, and the difference between groups was statistically significant (*P* < 0.0001) (Table 11).

**Figure 6:**
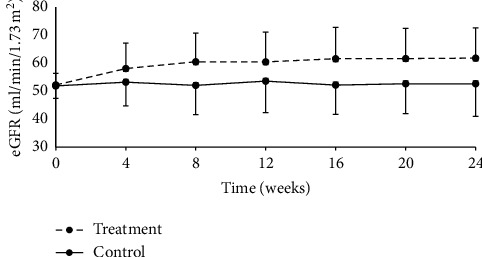
Change from baseline in eGFR over the 24-week follow-up period in the CKD stage 3a subgroup.

**Figure 7:**
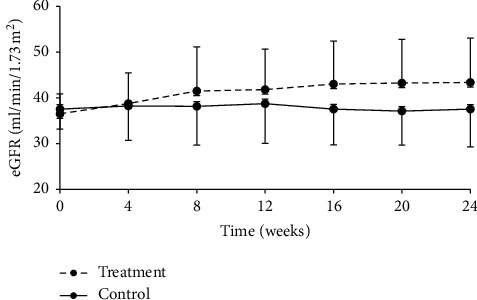
Change from baseline in eGFR over the 24-week follow-up period in the CKD stage 3b subgroup.

When eGFR data were further analyzed after stratification by CKD stages (3a or 3b), eGFR values increased from baseline in the treatment group, but no obvious change was observed in the control group. Difference in eGFR change between the two groups was statistically significant, and such trend persisted throughout the treatment (Tables 12–14, Figures 6 and 7).

### 3.4. Transfer in Stages of CKD Disease

**Figure 8:**
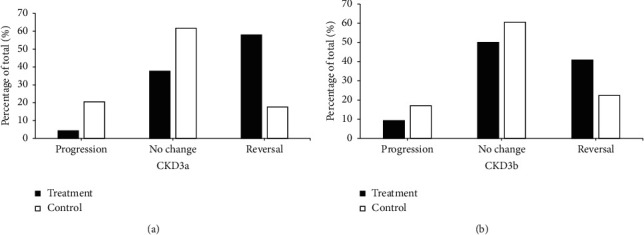
Evaluation and analysis of CKD stages 3a and 3b.

Among subjects with CKD stage 3a in the treatment group, the proportion of individuals progressing from stage 3a to stage 3b or 4 was 4.35%, 37.68% of them showed no change, and 57.97% of them were reversed to stage 2 or 1. The proportions of CKD stage 3a subjects showing progression, stabilization, and reversion were 20.59%, 61.76%, and 17.65%, respectively, in the control group. The differences were statistically significant. Among participants with CKD stage 3b in the treatment group, the proportion of individuals progressing from stage 3b to stage 4 was 9.21%, 50.00% of them showed no change, and 40.79% of them were reversed to stage 3a, 2 or 1. The proportions of CKD stage 3b subjects showing progression, stabilization, and reversion were 17.11%, 60.52%, and 22.37%, respectively, in the control group (Figure 8).

### 3.5. Safety and Adverse Events

There were no statistically significant differences between groups in laboratory parameters, either hematology, liver function, or electrolytes, after treatment (Table 15). During 24-week follow-up, the incidence of adverse events was 14.04% in the treatment group and 9.3% in the control group, without significant difference (Table 16). Two subjects in the treatment group, one experiencing 1 serious adverse event and the other suffering from benign meningioma and duodenal tumor (adenocarcinoma), were admitted to hospital. These serious adverse events were judged to have nothing to do with the study drug, by physicians. According to the analysis on adverse events (Table 17), the most prevalent events were mild abnormal liver function and mild elevation of blood potassium. However, overall incidence of adverse events was not significantly different between groups, suggesting that the study drug did not elevate the occurrences of adverse events.

## 4. Discussion

Our study indicated that Chinese herbal formula granules improved renal function as evidenced by decrease in serum creatinine and increase in eGFR in patients with CKD stage 3. 24-hour proteinuria was not reduced by TCM formula and was not significantly different between the two groups. The incidence of adverse events was not statistically different between groups either, indicating the safety of Chinese herbal formula granules.

Several factors may contribute to the progression of kidney damage, including hypertension, ageing, hemodynamic dysregulation, proteinuria, and high intake of dietary protein. Apart from kidney disease, proteinuria has been identified as a strong risk factor for CKD progression, and cardiovascular and all-cause mortality. Effective control over proteinuria may be renoprotective, and proteinuria is a crucial indicator for the measurement of treatment response in a variety of kidney diseases. Renin angiotensin-aldosterone system (RAS) blockers, glucocorticoids, and immunosuppressants are commonly used for primary glomerular diseases. Immunosuppressive therapies have been mainly adopted to treat patients with heavy proteinuria, but they are not entirely suitable for those with non-nephrotic-range proteinuria. Furthermore, long duration of treatment and high incidence of severe adverse effects limit the use of glucocorticoids and immunosuppressants. Therefore, in addition to RAS inhibitors, identifying other therapeutic agents would be necessary for patients with minor- to moderate-range proteinuria. TCM is widely regarded as a potential cost-effective alternative. The efficacy of Chinese medicine has been confirmed in some randomized controlled studies in recent years [9, 10]. However, studies on Chinese herbal compound formula are limited.

Measurement on proteinuria could predict renal outcomes, including dipstick urinalysis, urine albumin-to-creatinine ratio (ACR) or protein-to-creatinine ratio (PCR), and 24-hour urinary albumin or protein excretion. 24-hour urine collection for protein measurement is still considered to be the golden standard for measuring protein. ACR showed no superiority to PCR in predicting prognosis or detecting CKD in nondiabetic subjects [17]. Urine PCR, ACR, and 24-hour protein were reported to have equal utility to predict the doubling of serum creatinine, the commencement of renal replacement therapy, and all-cause mortality, according to a single-center retrospective cohort study [18]. In our study, due to heterogeneity in relevant standards across 9 hospitals, 24-hour proteinuria instead of ACR or PCR was adopted as a primary outcome to evaluate proteinuria. In the current study, Chinese herbal formula granules did not reduce proteinuria in patients with CKD stage 3. This might be ascribed to the short-term therapy and the fact that it was more difficult to lower proteinuria among patients with targeted CKD stage 3 which is more severe. It should be noted that changes in 24-hour proteinuria was much less in the TCM group than in control at every 4-week follow-up timepoint. So, the effectiveness of Chinese herbal formula granules on proteinuria needs to be further investigated.

Serum creatinine level is inversely correlated with GFR and is considered as an indirect marker of GFR. It can be used in estimation equations for GFR to approximate GFR. Notably, there was a gradual decline in serum creatinine in the TCM group. These results revealed that serum creatinine was obviously decreased and eGFR, calculated by the serum-based CKD-EPI equation, was significantly increased in the treatment group. Moreover, blood urea nitrogen remained unchanged. The accuracy of the eGFR equation is difficult to guarantee in a heterogeneous population. The CKD-EPI equation using creatinine, which is adopted widely, has such an accuracy that 80.6% of estimated GFR values are within 30% of measured GFR [19]. In day-to-day clinical practice, accuracy may be not necessary, and establishing an eGFR trend for an individual patient with CKD is probably more important. These outcomes demonstrated the efficacy of Chinese herbal formula granules in delaying the deterioration of kidney function in patients with CKD stage 3 for 6 months. This renal-protective function independent of proteinuria-lowering effects of TCM raises the question of how TCM compounds slow the progression of CKD.

Recently, accumulating clinical studies have focused on Chinese herbs for the treatment of CKD [20–22]. Since 2003, our group has been dedicated to characterizing the pathogenesis of CKD and to exploring the efficacy of TCM therapies [23, 24], and has proposed a complete TCM theory for the pathogenesis and treatment of CKD [25, 26]. As a result, several clinical trials and basic studies have been conducted to date [16, 27, 28]. The present study in patients with CKD stage 3 was the first prospective randomized controlled trial to evaluate clinical efficacy and safety of Chinese herbal formula compound which could tonify the kidney, clear away dampness, regulate circulation, and eliminate turbidity, according to TCM classic theory.

The studied Chinese herbal formula is consisted of 10 Chinese medicine herbs. According to TCM classic theory, *Astragali* (*Huangqi*), *Angelica sinensis* (*Danggui*), *Polygonati* (*Huangjing*), and *Achyranthis bidentatae* (*Niuxi*) could benefit qi, nourish yin, and tonify the kidney and spleen to nourish blood. *Astragali* (*Huangqi*) plus *Angelica sinensis* (*Danggui*) can tonify the kidney and benefit qi. *Astragali* (*Huangqi*) combined with *Polygonati* (*Huangjing*) could benefit qi and nourish yin. *Angelica sinensis* (*Danggui*) accompanied by *Achyranthis bidentatae* (*Niuxi*) could replenish blood and promote blood circulation. *Smilacis Glabrae* (*Tufuling*), *Serissa* (*Liuyuexue*), *Centella* (*Jixuecao*), *Polygoni cuspidati* (*Huzhang*), *Pyrrosiae* (*Shiwei*), and *Rhei* (*Dahuang*) all could clear away damp-heat, promote blood circulation to remove blood stasis, boost uresis, and alleviate strangury. Collectively, these 10 Chinese medicine herbs tonify the kidney, clear away dampness, regulate circulation, and eliminate turbidity. With the development of modern TCM pharmacology, therapeutic targets and effective constituents of herbs are identified, which fills important gaps in our knowledge on herbs. *Astragali* and *Angelica* have been reported to be able to delay the progression of renal diseases by improving local tissue perfusion, balancing vasoactive substances, and directly improving endothelial function [29]. *Polygoni cuspidate*, whose major ingredient is polydatin, inhibits the expression of intercellular adhesion molecule 1 (ICAM-1) and serum tumor necrosis factor-alpha (TNF-*α*) in the process of renal ischemia-reperfusion injury [30]. In addition, *Polygoni Cuspidati* could lower lipid level and relieve renal impairment [31, 32]. *Polygonati* regulates renal hemodynamics and slows the progression of CKD [31]. *Smilacis Glabrae* and *Serissae* can decrease serum uric acid and protect the kidney [33]. *Centella* ameliorates tubular interstitial fibrosis [34]. *Rhei* has been documented to delay the loss of renal function in both in vivo and in vitro studies [35]. By far, detailed mechanisms of the current TCM compound are still not completely understood. Possible mechanisms by which Chinese herbal formula granules preserve renal function may be ascribe to antirenal fibrosis, anti-inflammation, antioxidative stress, regulating microcirculation, and improving metabolism.

This clinical trial had some limitations. Firstly, the patients enrolled in this study had not been confirmed through renal biopsy. Secondly, proteinuria status varied among enrolled participants, even less than 2.0 g/d, and patients without proteinuria were recruited. Thirdly, we used 24-hour proteinuria and serum creatinine instead of the incidence of ESKD and cardiovascular disease as primary outcomes. Besides, short follow-up duration also represented a limitation. Additionally, ARBs were adopted to control blood pressure, but calculated dosages of ARBs were not recorded or compared between treatment and control groups. Given the function of ARBs on protecting the kidney, the application of ARBs might influence analysis results. Therefore, further investigations are required to improve our findings.

In conclusion, Chinese herbal formula granules could preserve renal function in patients with CKD stage 3, independent of proteinuria reduction, and could be employed as an effective and safe therapy for patients with CKD stage 3.

## Figures and Tables

**Table 1:** Constituents and dosage of one Chinese herbal formula granules.

Herb name	Dosage ratio in one prescription (%)
Astragali Radix (Huangqi)	6
Angelica Sinensis Radix Tostum (Danggui)	2
Polygoni Cuspidati Rhizoma et Radix (Huzhang)	3
Herba Serissae (Liuyuexue)	6
Smilacis Glabrae Rhizoma (Tufuling)	6
Achyranthis Bidentatae Radix (Niuxi)	2
Pyrrosiae Folium (Shiwei)	4
Rhei Radix et Rhizoma Praeparata (Dahuang)	1
Centellae Herba (Jixuecao)	6
Polygonati Rhizoma Praeparata (Huangjing)	4

**Table 2:** Participant disposition.

	Treatment group no. (%)	Control group no. (%)	Total no. (%)
Randomization	171 (100.00)	172 (100.00)	343 (100.00)
Safety analysis set (SS)	171 (100.00)	172 (100.00)	343 (100.00)
Full analysis set (FAS)	171 (100.00)	172 (100.00)	343 (100.00)
Dropouts during the study	21 (12.28)	22 (12.79)	43 (12.54)
Serious adverse events	2 (1.17)	0 (0.00)	2 (0.58)
Nonadherence to medication, e.g., discontinuation from study drug for more than 2 weeks without permission	0 (0.00)	2 (1.16)	2 (0.58)
Withdrawal by subject	16 (9.36)	17 (9.88)	33 (9.62)
Others (reasons)	3 (1.75)	3 (1.74)	6 (1.75)
Per-protocol set (PPS)	150 (87.72)	150 (87.21)	300 (87.46)

**Table 3:** Dropout rate and compliance in the enrolled participants.

Variable	Treatment group	Control group	Statistics	*P* value
Dropout			0.02 (CHISQ test)	0.8866
No	150 (87.72)	150 (87.21)		
Yes	21 (12.28)	22 (12.79)		
Compliance scores	92.57 ± 24.99	94.18 ± 22.67	−0.62 (grouped *T*-test)	0.5338

**Table 4:** Patient demographics and baseline characteristics (full analysis set).

Variable	Treatment group	Control group	Statistics	*P* value
Gender			2.68 (CHISQ test)	0.1017
Male	106 (61.99)	121 (70.35)		
Female	65 (38.01)	51 (29.65)		
Age (years)	51.89 ± 13.12	52.03 ± 12.62	−0.10 (grouped *T*-test)	0.9197
Disease duration (months)	59.92 ± 84.69	62.81 ± 77.05	−0.33 (grouped *T*-test)	0.7424
Systolic blood pressure (mmHg)	124.57 ± 7.24	123.84 ± 7.90	0.89 (grouped *T*-test)	0.3730
Diastolic blood pressure (mmHg)	76.94 ± 5.24	76.56 ± 6.12	0.61 (grouped *T*-test)	0.5408
24-hour proteinuria (g/24 h)	0.73 ± 0.61	0.72 ± 0.67	0.22 (grouped *T*-test)	0.8298
Urea nitrogen (mmol/L)	8.93 ± 3.26	8.89 ± 3.10	0.09 (grouped *T*-test)	0.9291
Serum creatinine (*μ*mol/L)	150.27 ± 37.12	147.56 ± 29.46	0.75 (grouped *T*-test)	0.4540
eGFR (ml/min/1.73 m^2^)	43.57 ± 9.29	44.37 ± 9.09	−0.81 (grouped *T*-test)	0.4171

**Table 5:** Change from baseline in 24-hour proteinuria over the 24-week follow-up period.

Time	Treatment group	Control group	Effect	*F* value	*P* value
Pretreatment (g/24 h)	0.72 ± 0.61	0.66 ± 0.58	Time effect	4.19	0.0009
Week 4 (g/24 h)	0.81 ± 0.83	0.72 ± 0.70	Difference between groups	3.10	0.0792
Week 8 (g/24 h)	0.82 ± 0.89	0.85 ± 1.16			
Week 12 (g/24 h)	0.84 ± 1.01	1.01 ± 1.59^*∗*^			
Week 16 (g/24 h)	0.81 ± 0.95	0.99 ± 1.39^*∗*^			
Week 20 (g/24 h)	0.87 ± 1.02	1.01 ± 1.47^*∗*^			
Week 24 (g/24 h)	0.97 ± 1.14	0.97 ± 1.25^*∗*^			

^*∗*^Compared with pretreatment, the difference was significant in the control group, *P* < 0.05.

**Table 6:** Change from baseline in serum creatinine over the 24-week follow-up period.

Time	Treatment group	Control group	Effect	*F* value	*P* value
Pretreatment (*μ*mol/L)	148.42 ± 35.90	147.26 ± 28.71	Time effect	3.11	0.0086
Week 4 (*μ*mol/L)	138.80 ± 31.62	148.06 ± 34.99	Difference between groups	54.69	0.0001
Week 8 (*μ*mol/L)	133.28 ± 30.95	150.22 ± 36.70			
Week 12 (*μ*mol/L)	132.59 ± 30.42	147.73 ± 39.02			
Week 16 (*μ*mol/L)	130.19 ± 29.79^*∗*^	150.50 ± 38.23			
Week 20 (*μ*mol/L)	130.08 ± 30.57^*∗*^	151.10 ± 36.18			
Week 24 (*μ*mol/L)	130.78 ± 32.55^*∗*^	149.12 ± 41.27			

^*∗*^Compared with pretreatment, the difference was significant in the treatment group, *P* < 0.05.

**Table 7:** Analysis of the concentration change in serum creatinine pre- and post-treatment.

Variable	Treatment group	Control group	Statistics	*P* value
Concentration change			−4.86 (grouped *T*′ test)	<0.0001
*N* (missing)	149 (1)	148 (2)		
Mean ± SD (*μ*mol/L)	−11.31 ± 15.02	3.74 ± 34.57		
Median (*μ*mol/L)	−13.15	0.48		
Min-max (*μ*mol/L)	−42.05–40.77	−96.73–320.82		

**Table 8:** Change from baseline in serum creatinine over the 24-week follow-up period in the CKD stage 3a subgroup.

Time	Treatment group	Control group	Effect	*F* value	*P* value
Pretreatment (*μ*mol/L)	130.27 ± 15.79	132.46 ± 21.82	Time effect	0.40	0.8511
Week 4 (*μ*mol/L)	120.93 ± 17.10	133.80 ± 23.82	Difference between groups	25.42	<0.0001
Week 8 (*μ*mol/L)	117.54 ± 19.61	136.63 ± 27.40			
Week 12 (*μ*mol/L)	117.74 ± 18.09	134.98 ± 32.59			
Week 16 (*μ*mol/L)	116.43 ± 18.70	136.87 ± 35.06			
Week 20 (*μ*mol/L)	116.13 ± 19.25	136.18 ± 30.23			
Week 24 (*μ*mol/L)	115.11 ± 19.18	136.21 ± 39.45			

**Table 9:** Change from baseline in serum creatinine over the 24-week follow-up period in the CKD stage3b subgroup.

Time	Treatment group	Control group	Effect	*F* value	*P* value
Pretreatment (*μ*mol/L)	165.75 ± 40.14	162.38 ± 27.96	Time effect	2.34	0.0403
Week 4 (*μ*mol/L)	156.07 ± 31.57	163.22 ± 38.99	Difference between groups	25.53	<0.0001
Week 8 (*μ*mol/L)	148.40 ± 31.59	164.48 ± 40.41			
Week 12 (*μ*mol/L)	147.72 ± 31.77	162.95 ± 37.74			
Week 16 (*μ*mol/L)	144.15 ± 30.74	166.01 ± 37.26			
Week 20 (*μ*mol/L)	143.73 ± 32.26	167.48 ± 36.37			
Week 24 (*μ*mol/L)	145.46 ± 34.02	163.64 ± 40.71			

**Table 10:** Change from baseline in eGFR over the 24-week follow-up period.

Time	Treatment group	Control group	Effect	*F* value	*P* value
Pretreatment (ml/min/1.73 m^2^)	44.04 ± 9.28	44.24 ± 8.75	Time effect	1.66	0.1422
Week 4 (ml/min/1.73 m^2^)	47.91 ± 12.56	45.19 ± 11.11	Difference between groups	33.04	<0.0001
Week 8 (ml/min/1.73 m^2^)	50.40 ± 13.73	44.69 ± 11.94			
Week 12 (ml/min/1.73 m^2^)	50.67 ± 13.48	45.66 ± 12.55			
Week 16 (ml/min/1.73 m^2^)	51.77 ± 13.85	44.41 ± 11.90			
Week 20 (ml/min/1.73 m^2^)	51.90 ± 13.68	44.39 ± 12.03			
Week 24 (ml/min/1.73 m^2^)	55.74 ± 50.82	44.46 ± 12.60			

**Table 11:** Analysis of the change of eGFR in pre- and post-treatment.

Variable	Treatment group	Control group	Statistics	*P* value
Change			7.40 (grouped *T*-test)	<0.0001
*N* (missing)	149 (1)	148 (2)		
Mean ± SD (ml/min/1.73 m^2^)	17.95 ± 21.86	0.04 ± 19.83		
Median (ml/min/1.73 m^2^)	17.93	−0.72		
Min-max (ml/min/1.73 m^2^)	−33.85–88.97	−58.75–63.27		

**Table 12:** Change from baseline in eGFR over the 24-week follow-up period in the CKD stage 3a subgroup.

Time	Treatment group	Control group	Effect	*F* value	*P* value
Pretreatment (ml/min/1.73 m^2^)	52.18 ± 4.14	51.85 ± 4.44	Time effect	2.08	0.0664
Week 4 (ml/min/1.73 m^2^)	58.06 ± 9.04	53.18 ± 8.45	Difference between groups	33.59	<0.0001
Week 8 (ml/min/1.73 m^2^)	60.45 ± 10.28	52.04 ± 10.41			
Week 12 (ml/min/1.73 m^2^)	60.43 ± 10.64	53.56 ± 11.24			
Week 16 (ml/min/1.73 m^2^)	61.54 ± 11.18	52.17 ± 10.48			
Week 20 (ml/min/1.73 m^2^)	61.60 ± 10.82	52.61 ± 10.65			
Week 24 (ml/min/1.73 m^2^)	61.77 ± 10.78	52.63 ± 11.63			

**Table 13:** Change from baseline in eGFR over the 24-week follow-up period in the CKD stage 3b subgroup.

Time	Treatment group	Control group	Effect	*F* value	*P* value
Pretreatment (ml/min/1.73 m^2^)	36.53 ± 4.36	37.53 ± 4.34	Time effect	3.4	0.0043
Week 4 (ml/min/1.73 m^2^)	38.76 ± 6.68	38.22 ± 7.50	Difference between groups	23.68	<0.0001
Week 8 (ml/min/1.73 m^2^)	41.49 ± 9.64	38.16 ± 8.49			
Week 12 (ml/min/1.73 m^2^)	41.80 ± 8.85	38.73 ± 8.65			
Week 16 (ml/min/1.73 m^2^)	43.01 ± 9.40	37.55 ± 7.81			
Week 20 (ml/min/1.73 m^2^)	43.23 ± 9.54	37.11 ± 7.42			
Week 24 (ml/min/1.73 m^2^)	43.37 ± 9.66	37.53 ± 8.23			

**Table 14:** Change from baseline in urea nitrogen over the 24-week follow-up period.

Time	Treatment group	Control group	Effect	*F* value	*P* value
Pretreatment (mmol/L)	8.75 ± 3.06	8.69 ± 2.69	Time effect	0.25	0.9388
Week 4 (mmol/L)	8.90 ± 2.68	8.24 ± 2.76	Difference between groups	4.26	0.0399
Week 8 (mmol/L)	9.02 ± 2.85	8.25 ± 2.61			
Week 12 (mmol/L)	9.12 ± 2.75	8.22 ± 2.40			
Week 16 (mmol/L)	8.94 ± 2.73	8.24 ± 2.25			
Week 20 (mmol/L)	8.93 ± 2.72	8.34 ± 2.38			
Week 24 (mmol/L)	8.76 ± 2.67	8.40 ± 2.54			

**Table 15:** Change from baseline in safety outcomes over the 24-week follow-up period.

Variable	Treatment group	Control group	*P* value time effect	*P* value group comparison
White blood cell (×10^12^/L)				
Pretreatment	6.49 ± 2.16	6.49 ± 1.81	0.2003	0.9694
Week 24	6.53 ± 1.99	6.51 ± 1.86		
Red blood cell (×10^12^/L)				
Pretreatment	4.40 ± 0.54	4.53 ± 0.62	0.0842	0.1398
Week 24	4.46 ± 0.56	4.69 ± 0.64		
Platelet count (×10^12^/L)				
Pretreatment	178.23 ± 49.27	190.31 ± 51.67	0.2136	0.2415
Week 24	192.03 ± 53.51	197.12 ± 53.75		
Albumin (g/L)				
Pretreatment	42.92 ± 3.92	42.87 ± 6.03	0.2117	0.3561
Week 24	44.28 ± 3.01	43.16 ± 3.90		
ALT (IU/L)				
Pretreatment	26.60 ± 15.62	23.91 ± 12.27	0.8650	0.2324
Week 24	26.52 ± 15.46	26.03 ± 11.39		
AST (IU/L)				
Pretreatment	25.83 ± 13.00	23.33 ± 10.15	0.6925	0.5820
Week 24	26.14 ± 12.69	23.72 ± 8.72		
Blood potassium (mmol/L)				
Pretreatment	4.38 ± 0.62	4.34 ± 0.65	0.4012	0.8847
Week 24	4.58 ± 0.59	4.33 ± 0.53		

**Table 16:** Analysis of adverse events.

Variable	Treatment group (*n*, %)	Control group (*n*, %)	Statistics	*P* value
Adverse event			1.86 (CHISQ test)	0.1721
Total (missing)	171 (0)	172 (0)		
No	147 (85.96)	156 (90.70)		
Yes	24 (14.04)	16 (9.30)		

**Table 17:** Analysis of adverse events by frequency.

Item	Treatment group			Control group		
	No. of patients with event	No. of events	Event rate (%)	No. of patients with event	No. of events	Event rate (%)
Abnormal liver function tests	8	8	4.55	8	10	4.47
Mild	5	5	2.84	6	8	3.35
Moderate	3	3	1.70	2	2	1.12
Abnormal hematology	4	6	2.27	2	3	1.12
Mild	3	5	1.70	0	0	0.00
Moderate	1	1	0.57	3	5	1.67
General Clinical Symptoms and Discomforts	0	0	0.00	1	3	0.56
Mild	2	2	1.14	1	3	0.56
Urinary tract infection	1	1	0.57	0	0	0.00
Moderate	1	1	0.57	0	0	0.00
Electrolyte disturbances	9	11	5.11	2	2	1.12
Mild	8	8	4.55	2	2	1.12
Moderate	1	3	0.57	0	0	0.00
Tumor	1	1	0.57	0	0	0.00
Benign	2	2	1.14	0	0	0.00
Malignant	0	0	0.00	0	0	0.00

## Data Availability

All data generated or analyzed during this study are included in this article.

## References

[1] Webster A. C., Nagler E. V., Morton R. L., Masson P. (2017). Chronic kidney disease. *The Lancet*.

[2] Barsoum R. S. (2006). Chronic kidney disease in the developing world. *New England Journal of Medicine*.

[3] Wang S., Chen R., Liu Q., Shu Z., Zhan S., Li L. (2015). Prevalence, awareness and treatment of chronic kidney disease among middle-aged and elderly: the China health and retirement longitudinal study. *Nephrology*.

[4] Liyanage T., Ninomiya T., Jha V. (2015). Worldwide access to treatment for end-stage kidney disease: a systematic review. *The Lancet*.

[5] Liu Z.-H. (2013). Nephrology in China. *Nature Reviews Nephrology*.

[6] Turner J. M., Bauer C., Abramowitz M. K., Melamed M. L., Hostetter T. H. (2012). Treatment of chronic kidney disease. *Kidney International*.

[7] Qaseem A., Hopkins R. H., Sweet D. E., Starkey M., Shekelle P. (2013). Screening, monitoring, and treatment of stage 1 to 3 chronic kidney disease: a clinical practice guideline from the American college of physicians. *Ann Intern Med*.

[8] Levey A. S., De Jong P. E., Coresh J. (2011). The definition, classification, and prognosis of chronic kidney disease: a KDIGO controversies conference report. *Kidney International*.

[9] Chen Y., Deng Y., Ni Z. (2013). Efficacy and safety of traditional Chinese medicine (shenqi particle) for patients with idiopathic membranous nephropathy: a multicenter randomized controlled clinical trial. *American Journal of Kidney Diseases*.

[10] Zhang L., Li P., Xing C.-Y. (2014). Efficacy and safety of *Abelmoschus manihot* for primary glomerular disease: a prospective, multicenter randomized controlled clinical trial. *American Journal of Kidney Diseases*.

[11] Zou C., Lu Z.-Y., Wu Y.-C. (2013). Colon may provide new therapeutic targets for treatment of chronic kidney disease with Chinese medicine. *Chinese Journal of Integrative Medicine*.

[12] Chen Y., Cai G., Sun X., Chen X. (2016). Treatment of chronic kidney disease using a traditional Chinese medicine, flos abelmoschus manihot (linnaeus) medicus (malvaceae). *Clinical and Experimental Pharmacology and Physiology*.

[13] Guan Y., Wu X.-X., Duan J.-L. (2015). Effects and mechanism of combination of rhein and danshensu in the treatment of chronic kidney disease. *The American Journal of Chinese Medicine*.

[14] Chen J., Sun W., Wei L., Liu X., Yuan F., Zhou D. (2013). Effect of traditional Chinese medicine differential treatment on glomerular filtration rate and urine protein in patients with stage 3 chronic kidney disease. *Journal of Traditional Chinese Medicine*.

[15] Wang X., Sun W. (2013). Clinical study of treatment of chronic kidney disease 4 phase with tonifying kidney clearing heat and draining damp activating blood plus tripterygium glycosides tablets. *Journal of Liaoning University of Traditional Chinese Medicine*.

[16] Zhang L., Zhou D., Wei M., Zhao J., Gao K. (2010). Clinical study of tonifying kidney and regulating and dispersing mudy in 40 patients with early and middle stage CKD. *Jiangsu Journal of Traditional Chinese Medicine*.

[17] Johnson D. W., Jones G. R. D., Mathew T. H. (2012). Chronic kidney disease and measurement of albuminuria or proteinuria: a position statement. *Medical Journal of Australia*.

[18] Methven S., MacGregor M. S., Traynor J. P., Hair M., O’Reilly D. S. J., Deighan C. J. (2011). Comparison of urinary albumin and urinary total protein as predictors of patient outcomes in CKD. *American Journal of Kidney Diseases*.

[19] Levin A., Stevens P. E., Bilous R. W. (2013). Kidney disease: improving global outcomes (KDIGO) CKD work group: KDIGO 2012 clinical practice guideline for the evaluation and management of chronic kidney disease. *Kidney International Supplements*.

[20] Fang Y. Q., Lu Y., Wang Y. J. (2012). Efficiency of benazepril combined with wind dispelling and dampness removing Chinese herbs on stage 3 chronic kidney disease with wind-dampness syndrome: a prospective study. *Zhongguo Zhong Xi Yi Jie He Za Zhi*.

[21] Ma Z., Peng W., Ni Z. (2016). Clinical effect of jianpi qinghua formula on patients with spleen deficiency and dampness-heat due to chronic kidney disease (stage III). *Chinese Journal of Traditional Chinese Medicine Pharmacy*.

[22] Tracy E. M., Wang Y., He L. (2013). Clinical research of traditional Chinese medicine combined with renal protective effects of traditional Chinese medicine formula for patients with chronic kidney disease stage 3: a multi-center, prospective, double-blinded, randomized controlled trials of 315 cases. *World Chines Medicine*.

[23] Sun W., Gao K., Zhou D. (2005). Analysis on combined therapy of tonifying the kidney removing dampness and activating blood circulation for treatment of 22 cases of focal and segmental glomerular sclerosis. *Chinese Journal of Integrated Traditional and Western Nephrology*.

[24] Sun W., Gao K., Zhou D. (2005). Clinical observation on combined therapy of tonifying the kidney removing dampness and activating blood circulation for treatment of 64 cases of focal and segmental glomerular sclerosis and IgA nephropathy. *Journal of Traditional Chinese Medicine*.

[25] Chen J., Gao K., Sun W. (2007). Reveals the pathogenesis of chronic kidney disease based on the theory of kidney deficiency and damp stasis. *Liaoning Journal of Traditional Chinese Medicine*.

[26] Sun W. (2011). On essence of kidney reinforcement and blood activation in treatment of chronic nephropathy. *Jiangsu Journal of Traditional Chinese Medicine*.

[27] Tu Y., Gu L., Chen D. (2017). Rhein inhibits autophagy in rat renal tubular cells by regulation of AMPK/mTOR signaling. *Scientific Reports*.

[28] Zhang L.-Q., Li W.-M., Sun W. (2012). Effect of yishen huayu fang on kidney tissue e-cadherin expression in unilateral ureter ligation in rats. *Journal of Traditional Chinese Medicine*.

[29] Song J. Y., Meng L. Q., Li X. M. (2008). Therapeutic application and prospect of Astragalus membranaceus and Angelica sinensis in treating renal microvascular lesions. *Zhongguo Zhong Xi Yi Jie He Za Zhi*.

[30] Li J., Li Y. (2010). The protective effect of polygonum and polydatin preconditioning on renal ischemia reperfusion injury in rats. *Global Traditional Chinese Medicine*.

[31] Fu X., Fu Z. (2012). Effects of polygonatum on hemodynamics of rats with chronic renal failure. *Chinese Archives of Traditional Chinese Medicine*.

[32] Krishnakumari S., Priya K. (2006). Hypolipidemic efficacy of *Achyranthes aspera* on lipid profile in sesame oil fed rats. *Ancient Science of Life*.

[33] Guo S., Zhang W., Zhang Y., Liu M., Liu L., Du X. (2011). Preventive and therapeutic effects of glabrous greenbrier rhizome on hyperuricemia and renal injury in rats. *Progress in Modern Biomedicine*.

[34] Zhang Z., Zhao L., Wang B., Wang S. (2008). Effects of *Centella asiatica* on expression of connective colledge tissue growth factor in UUO rats. *Chinese Journal of Integrated Traditional and Western Nephrology*.

[35] Li L. S., Liu Z. H. (1991). Clinical and experimental studies of rheum on preventing progression of chronic renal failure. *Zhong Xi Yi Jie He Za Zhi*.

